# ExactSearch: a web-based plant motif search tool

**DOI:** 10.1186/s13007-016-0126-6

**Published:** 2016-04-28

**Authors:** Chathura Gunasekara, Avinash Subramanian, Janaki Venkata Ram Kumar Avvari, Bin Li, Su Chen, Hairong Wei

**Affiliations:** School of Forest Resources and Environmental Science, Michigan Technological University, Houghton, MI 49931 USA; Department of Computer Science, Michigan Technological University, Houghton, MI USA; State Key Laboratory of Forest Genetics and Tree Breeding, Northeast Forestry University, Harbin, Heilongjiang 150040 People’s Republic of China; Life Science and Technology Institute, Michigan Technological University Houghton, Michigan, MI 49931 USA

## Abstract

**Background:**

Plant biologists frequently need to examine if a sequence motif bound by a specific transcription or translation factor is present in the proximal promoters or 3′ untranslated regions (3′ UTR) of a set of plant genes of interest. To achieve such a task, plant biologists have to not only identify an appropriate algorithm for motif searching, but also manipulate the large volume of sequence data, making it burdensome to carry out or fulfill.

**Result:**

In this study, we developed a web portal that enables plant molecular biologists to search for DNA motifs especially degenerate ones in custom sequences or the flanking regions of all genes in the 50 plant species whose genomes have been sequenced. A web tool like this is demanded to meet a variety of needs of plant biologists for identifying the potential gene regulatory relationships. We implemented a suffix tree algorithm to accelerate the searching process of a group of motifs in a multitude of target genes. The motifs to be searched can be in the degenerate bases in addition to adenine (A), cytosine (C), guanine (G), and thymine (T). The target sequences to be searched can be custom sequences or the selected proximal gene sequences from any one of the 50 sequenced plant species. The web portal also contains the functionality to facilitate the search of motifs that are represented by position probability matrix in above-mentioned species. Currently, the algorithm can accomplish an exhaust search of 100 motifs in 35,000 target sequences of 2 kb long in 4.2 min. However, the runtime may change in the future depending on the space availability, number of running jobs, network traffic, data loading, and output packing and delivery through electronic mailing.

**Conclusion:**

A web portal was developed to facilitate searching of motifs presents in custom sequences or the proximal promoters or 3′ UTR of 50 plant species with the sequenced genomes. This web tool is accessible by using this URL: http://sys.bio.mtu.edu/motif/index.php.

## Background

Plant biologists often need to examine if a given binding motif recognized by a specific transcription or translation factor is present in the proximal promoters or 3′ untranslated regions (3′ UTR) of a set of plant genes. To do this, biologists often need to download the flanking sequences of potential candidate target genes, and then identify appropriate algorithms to search for the given motifs. This can become constrained when many motifs have to be searched in a multitude of target sequences. Such an approach often becomes infeasible when degenerate motifs are encountered. To search for many motifs in a large number of target sequences, it is necessary to identify an efficient software, set up a computational environment, and prepare the required input files, and then extract essential information from outputs, during which, some fundamental bioinformatics skills are demanded. For example, preparation of the input files containing the proximal regions of promoters and 3′ UTRs, and write programs to parse and extract the outputs. All these can pose a challenge to biologists. To facilitate motif search and analysis, we have implemented an adequately efficient suffix tree-based search algorithm and incorporated it into a web application, which automated the whole process and hided the actual complexity from users. In addition to implementing the algorithm, we have downloaded and stored the flanking sequences of all genes from 50 genome-sequenced plant species in our web portal for retrieving [1]. In our web application, a user can select which of 50 species and which portions of flanking regions to search. We also made the gene identifiers and annotation of all 50 species available in our web portal allowing users to download and extract a list of gene identifiers for motif search.

## Implementation

Currently, there are some algorithms for substring search [2–4] but none of these theoretical studies has been made available to users as a web portal that has search capability of the degenerate motifs in large quantity of sequences from 50 plant species. In our ExactSearch web portal, users have two options to do exact motif search: (1) search motifs in users’ custom sequences by uploading a file containing motifs and a file containing target sequences of their own to search. (2) Search motifs in the flanking sequences of genes in one of the 50 plant species by entering a number of motifs and a list of gene IDs. In this circumstance, the web server helps retrieve the appropriate sequences from the database. The degenerate motifs should be given in FASTA format with IUPAC notation. We have downloaded the gene identifiers and annotations of all 50 the plant species from Phytozome, the Plant Comparative Genomics portal of the Department of Energy’s Joint Genome Institute (http://phytozome.jgi.doe.gov). The flanking sequences, namely 1.0, 1.5 and 2.0 kb upstream and 0.6 kb downstream with respect to the coding regions, of all genes in the 50 plant species were retrieved from Phytozome through the BioMart tool provided on webpage of phytozome (https://phytozome.jgi.doe.gov/pz/portal.html) without considering possible overlaps. All sequences were stored in our web server in four different groups for motif search. Although the lengths of 3′ UTRs in plant species are usually less than 300 nucleotides [5, 6], we chopped 600 nucleotides in case some subsets of genes in the genomes may have unusual long 3′ UTRs, needless to say that the results generated from 300 to 600 nucleotides downstream the STOP codons can always be ignored if users are not interested in this region.

## Results and discussion

### Suffix-tree based search algorithm and implementation

**Fig. 1:**
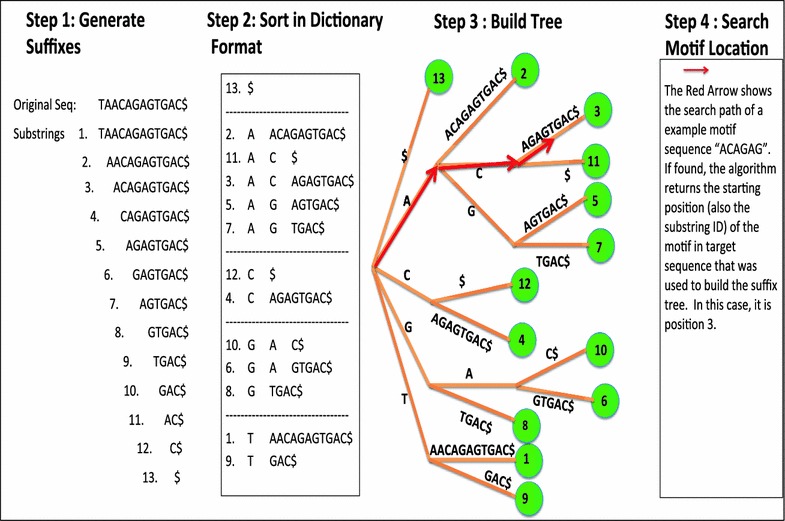
An illustration of the procedure and mechanism of suffix tree algorithm in searching for motifs

To find a group of motifs in a multitude of target sequences, we implemented an efficient string-searching algorithm called the suffix tree [7] (Fig. 1). As the first step towards locating the motifs in the given gene sequence, we generated all suffixes of a given target DNA sequence and stored them into a suffix tree, which is illustrated in Fig. 1. In brief, each target sequence was used to generate n-1 substrings, by chopping off one base each time from 5′ terminals. Then we started to store these substrings onto a tree as a dictionary (Fig. 1), where each node contains information about other sub sequent nodes and strings. When the first character of a substring was not present at the first level of suffix tree, a node with this character immediately under the root was created, and then the rest of substring was stored as a string immediately under the current node. If the first *i* ($$ i > 0) $$ characters in a to-be-stored substring matched characters sequentially along a path in the tree, a new node was created under the current node *i* with the *i* + 1 character being stored in this new node, and the rest of substring was stored in a node under this new node. In the other scenario, when the first *i* characters in a to-be-stored substring matched a path in the suffix tree, and the *i* + 1 character in the substring matched the first character of a sub-substring that was already stored at the level of the *i* + 1, a new node was created at the level of *i* + 1, where the common character of the substring and sub-substring was stored. Then the rest part of substring and the sub-substring were continuously compared and stored in the same nodes until two different characters from substring and the sub-substring were reached, then the rest parts of substring and sub-substring were stored in two different branches under the last new node created. Once a tree of a given target sequence was constructed in memory, another function worked through searching for a motif by navigating through tree structure in character-by-character fashion. If a motif has a length of m characters, searching for a motif is a facile task with O(m) running time. We only need to compare the motif with the sequence represented by a depth of m in the suffix tree. If the function passed though the entire motif sequence, the function returned true with the given number of the particular path (shown in green nodes), which is the starting location of the motif sequence in the target sequence. If users search for a degenerate motif (IUPAC standard), all the possible motifs are generated and searched individually. In IUPAC standard, each of character represents several possible bases. W: A or T, S: C or G, M: A or C, K: G or T, R: A or G, Y: C or T, B: C, G or T, D: A, G, or T, H: A, C, or T, V: A, C, or G and N: A, C, G, or T.

### Motifs in position probability matrix (PPM) search with motifLocator and background models

To facilitate the search for motifs represented by position probability matrix, we implemented an easy to use web interface for the motifLocator [8, 9]. A background model was created for each length of proximal promoter regions, or the 3′ UTRs of all genes in each species. For searching a motif represented by a PPM, users need to select a background model and the motif file that contains at least one motif in PPM format.

### Comparison with existing software

ExactSearch, the web-based tool we developed is mainly for searching degenerate motifs in the proximal gene sequences of 50 genome-sequenced plant species. The users have the options to either submit their own target sequences or select from 50 plant species. The other existing web portal, such as MEME suite (http://meme-suite.org), is a novel motif discovery tool. The discovered motifs are represented as position-dependent letter-probability matrices that describe the probability of each possible letter at each position in the pattern. For motif search tools enclosed in the MEME web portal, such as FIMO [10], MAST [11], and MCAST [12], the input motifs must be only in MEME motif format, namely, position-dependent letter-probability matrices. Another tool, GLAM2Scan [13], enclosed in MEME package can search for gapped local alignments of motifs using an output format of MEME suite. However, all these tools are incapable of taking degenerate sequences in fasta format as a motifs input file. Therefore, ExactSearch is a different tool in comparison to the above-mentioned web-based tools. Our web-tool caters to a different need of finding the already known degenerate motif sequences in a large number of plant proximal gene sequences in a very efficiently manner. In addition, our web-based tool harbors plant proximal gene sequences of 50 species for users to select, allowing extensive search for the known motifs sequences.

## Conclusion

A user-friendly web-based tool was developed to facilitate the searching of DNA motifs in either custom sequences or the proximal promoters or 3′ UTR of 50 genome-sequenced plant species using suffix-tree algorithm. To operate the tool, the users only need a web browser and have access to an electronic mail account. For exact motif search, when a job is completed, the user receives an attachment file (<25 MB), or a download link of a big size file (>25 MB). For matrix motif search, the user receives a download link of a compressed directory.
